# A Novel *Pseudoalteromonas xiamenensis* Marine Isolate as a Potential Probiotic: Anti-Inflammatory and Innate Immune Modulatory Effects against Thermal and Pathogenic Stresses

**DOI:** 10.3390/md19120707

**Published:** 2021-12-15

**Authors:** Withanage Prasadini Wasana, Amal Senevirathne, Chamilani Nikapitiya, Tae-Yang Eom, Youngdeuk Lee, Jong-Soo Lee, Do-Hyung Kang, Chulhong Oh, Mahanama De Zoysa

**Affiliations:** 1 Department of Preventive Medicine, College of Veterinary Medicine, Chungnam National University, Yuseong-gu, Daejeon 34134, Korea; wasanaprasadini@o.cnu.ac.kr (W.P.W.); amal.senevirathne@cnu.ac.kr (A.S.); chamilani14@cnu.ac.kr (C.N.); jongsool@cnu.ac.kr (J.-S.L.); 2 Jeju Marine Research Center, Korea Institute of Ocean Science and Technology (KIOST), Gujwa-eup, Jeju City 63349, Korea; eomsun@kiost.ac.kr (T.-Y.E.); lyd1981@kiost.ac.kr (Y.L.); dohkang@kiost.ac.kr (D.-H.K.); 3 Department of Ocean Science, University of Science and Technology, 217, Gajeong-ro, Yuseong-gu, Daejeon 34113, Korea

**Keywords:** probiotic, *Pseudoalteromonas xiamenensis*, zebrafish, mucin, *Edwardsiella piscicida*

## Abstract

A marine bacterial strain was isolated from seawater and characterized for it beneficial probiotic effects using zebrafish as a model system. The strain was identified by morphological, physiological, biochemical, and phylogenetic analyses. The strain was most closely related to *Pseudoalteromonas xiamenensis* Y2, with 99.66% similarity; thus, we named it *Pseudoalteromonas xiamenensis* S1131. Improvement of host disease tolerance for the *P. xiamenensis* isolate was adapted in a zebrafish model using *Edwardsiella piscicida* challenge. The larvae were pre-exposed to *P. xiamenensis* prior to *E. piscicida* challenge, resulting in a 73.3% survival rate compared to a 46.6% survival for the control. The treated larvae tolerated elevated temperatures at 38 °C, with 85% survival, compared to 60% survival for the control. Assessment of immunomodulatory responses at the mRNA level demonstrated the suppression of pro-inflammatory markers *tnfα* and *il6*, and upregulation of heat shock protein *hsp90* and mucin genes. The same effect was corroborated by immunoblot analysis, revealing significant inhibition of Tnfα and an enhanced expression of the Hsp90 protein. The antibacterial activity of *P. xiamenensis* may be related to mucin overexpression, which can suppress bacterial biofilm formation and enhance macrophage uptake. This phenomenon was evaluated using nonstimulated macrophage RAW264.7 cells. Further studies may be warranted to elucidate a complete profile of the probiotic effects, to expand the potential applications of the present *P. xiamenensis* isolate.

## 1. Introduction

Environmental damage and associated diseases are a serious challenge for mankind in the present century. The demand for innovative approaches is higher than ever to cope with these issues in the more resource-limited and fragile environment of the modern era [1]. Excessive use of antibiotics, antiseptics, and chemotherapeutic agents have major environmental implications, and have resulted in the development of multidrug-resistant bacteria [2]. The accumulation of antibiotic and chemical residues in the environment is affecting the natural equilibrium of the flora and fauna, for which the potential outcome has not been fully elucidated [3,4,5]. Therefore, the use of chemical agents has to be minimized, unless absolutely essential. In recent studies, probiotic bacterial species have also demonstrated their antimicrobial abilities against various pathogens. The modes of action of probiotics are quite different to the activity of chemicals such as antibiotics. However, probiotic species hold the promise to be used alongside chemical antimicrobial approaches, thus limiting the actual usage of chemical substances. In the present context, the discovery of biological approaches such as probiotic bacterial strains for disease control can be highly desirable due to their lesser impact on the environment, as well as a multitude of other beneficial outcomes. According to the World Health Organization definition, probiotics are live microorganisms that, when administered in adequate amounts, confer a health benefit to the host [6]. These benefits include immunity improvement [7], disease control [8,9], stress tolerance, digestion, and food absorption improvement [8]. The underlying mechanisms of many probiotics have not been fully elucidated. However, several mechanisms have been proposed, including modification of the gut microbiota, secretion of antimicrobial substances, strengthening of the gut epithelial barrier, immune modulation, and competitive adherence to the epithelium leading to pathogen exclusion [10]. Due to the diversity of probiotic species, their underlying mechanisms can also be significantly varied, hence they are difficult to generalize.

Applications of probiotic bacteria can be extended beyond their use in human subjects. These bacteria can be effectively utilized for productivity improvements in aquatic animals as well [11,12]. Several bacterial isolates have been proven to be effective for disease prevention in aquatic animals. For instance, a *Pseudoalteomonas* sp. isolated from the intestinal tract of Atlantic cod (*Gadus morhua*) was found to be antagonistic against pathogenic *Vibrio anguillarum* [13]. In another study, *Pseudalteromonas* sp. FC228, enriched in sea cucumber (*Apostuchopus japonicas*), demonstrated immune modulation and resistance against *V. spledidus* infection [14]. It was also reported that bacteria isolated from marine environments tend to possess more stable enzymes and active compounds than terrestrially isolated strains [15]. Hence, marine bacteria may possess a higher functional stability to survive in harsh environments. The ability of probiotic strains to tolerate the gastrointestinal environment is also a matter of concern. Hence, a search for suitable marine isolates for probiotic applications can reasonably be justified. 

In this study, we isolated a marine *Pseudoalteromonas* sp. and evaluated it as a potential probiotic candidate to stimulate the host immune system to fight disease, and to enhance environmental fitness. For initial characterization, species-level identification, determination of optimal growth conditions, augmentation of disease and heat-stress tolerance, and the induction of immunological markers and safety aspects were performed using the zebrafish larval model as an appropriate animal model for a marine-isolated bacterium. We refer to the original isolate *Pseudoalteromonasxiamenensis* S1131 as *P. xiamenensis* in this report. The augmentation of disease resistance was demonstrated in an *Edwardsiella piscicida* challenge experiment in zebrafish larvae, after co-exposure of the larvae in a *P. xiamenensis-*inoculated environment. Exposure to *P. xiamenensis* significantly improved larval resistance to pathogenic *E. piscicida,* and conferred increased tolerance to higher environmental temperatures than the non-treated control group. We also demonstrate that the *P. xiamenensis* isolate is safe and non-pathogenic to sensitive zebrafish larvae. Furthermore, molecular characterization studies revealed a modulation of cytokines and heat shock proteins, which mirrors the underlying mechanisms of its probiotic effects. Broad spectrum physiological and biochemical characterizations were conducted to identify biochemical capabilities that might differentiate *P. xiamenensis* from other related bacteria. Based on our observations, we hypothesized that mucin can be a potential molecular target that orchestrates host innate and adaptive antibacterial defense upon *P. xiamenensis* treatment.

The effects of the *P. xiamenensis* marine isolate on disease control and the enhancement of environmental tolerance may pave the way to develop a usable probiotic strain, which could be useful for disease prevention and control in commerciallyimportant aquatic animals. Further studies may be warranted to fully characterize its probiotic effect, such as the examination of other productivity aspects expected from a conventional probiotic.

## 2. Results

### 2.1. Identification, Morphological, and Physiological Characterization

**Figure 1:**
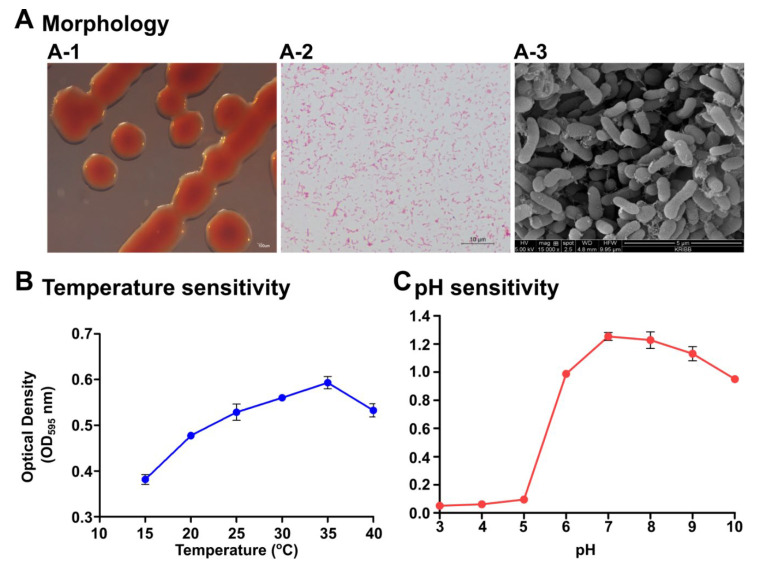
Morphological and physiological characterization of *Pseudoalteromonas xiamenensis* S1131. (**A**) Colony morphology on marine agar (**A-1**), Gram staining (**A-2**), and scanning electron microscopy (**A-3**) demonstrating the morphological appearance of the *P. xiamenensis* isolate. Scale bars indicate 100 µm, 10 µm, and 5 µm length for colony morphology, Gram staining, and SEM, respectively. Physiological characterization determining the optimum temperature (**B**) and pH (**C**) conditions. Cultures were incubated for 24 h under specified temperatures and pH conditions and the absorbance measurements were taken at OD_595_.

**Figure 2:**
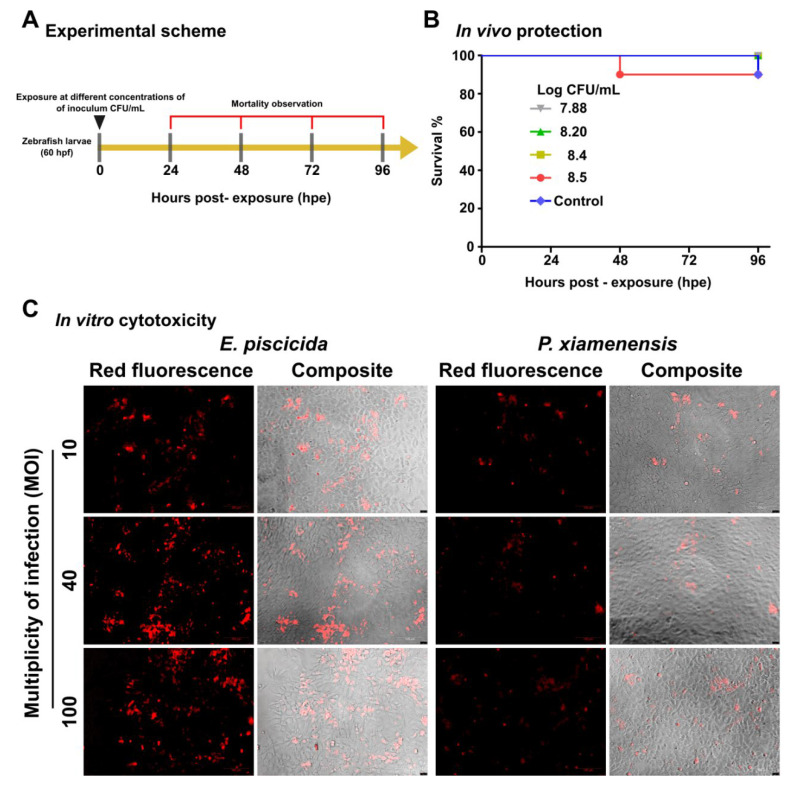
Safety assessments of the probiotic isolate, *P. xiamenensis,* and pathogenic strain, *E. piscicida*. In vivo safety assessment was conducted by exposing zebrafish larvae to different concentrations of *P. xiamenensis* in egg water. (**A**) Schematic representation for the experimental schedule of *P. xiamenensis* exposure to the zebrafish larvae. (**B**) Kaplan−Meier survival curves of zebrafish larvae upon exposure to the different concentrations of *P. xiamenensis.* (**C**) In vitro cytotoxicity assay. Fathead minnow (FHM) fish epithelial cells were either exposed to *P. xiamenensis* probiotic isolate, or *E. piscicida* pathogenic strain. After 1 h of incubation, cells were washed with PBS and non-internalized bacteria were eliminated by gentamycin treatment. The cells were replenished with complete growth media and further incubated for 12 h. After 12 h incubation, the cells were subjected to propidium iodide (PI) staining and observed under a fluorescent microscope. Images were taken at 200 × magnification.

**Figure 3:**
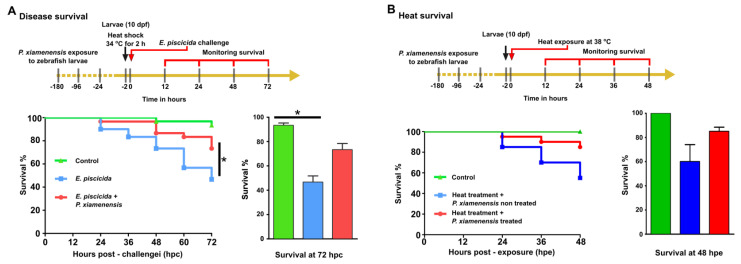
Effect of *P. xiamenensis* on disease resistance and heat stress survival. (**A**) Treatment with *P. xiamenensis* was evaluated for disease resistance enhancement against fish-pathogenic *E. piscicida*. After *P. xiamenensis* treatment, zebrafish larvae were challenged with *E. piscicida*. Mortality was recorded for 72 h post-exposure (hpe) and the relative percent survival was determined. Asterisk (*) indicates a significant difference in comparison to the non-infected control. (**B**) Enhancement of heat resistance was evaluated for zebrafish after *P. xiamenensis* treatment at 28 °C followed by heat stress exposure at 38 °C for 48 h. The mortality of fish larvae was evaluated. Statistical significance was set at *p* < 0.05.

The strain S1131 was originally isolated from seawater collected from Chuuk state, Federated States of Micronesia. The isolated strain was tested for microscopic and physiological characterization. Colonies were uniform, watery, and red in appearance, with an approximate size of 2–3 mm in diameter after 24 h incubation at 28 °C (Figure 1(A-1)). The bacterium was negative for Gram staining (Figure 1(A-2)) and was rod shaped (Figure 1(A-3)). Secretion of the extracellular matrix was also evident. We determined the optimum temperature and pH for *P. xiamenensis* growth, from 15 to 40 °C, and pH 3–10, respectively, using a marine broth medium. The ideal growth of the bacterium was found to be 35 °C (Figure 1B) and at a neutral pH level of 7 (Figure 1C). In addition, diffusion experiments revealed that S1131 does not produce any toxic substances harming other bacteria species, such as *Enterococcus faecalis*, *Staphylococcus epidermidis*, *Pseudomonas aeruginosa,* *E. piscicida*, and *Aeromonas hydrophila*. The molecular level confirmation of strain S1131 was conducted by 16S rRNA sequencing using 27F and 1492R standard primers. The nucleotide sequence was 1471 bp, and closest to *P*. *xiamenensis* Y2 (type strain), with a 99.66% similarity in the phylogenetic analysis (Figure S1). Therefore, we named our isolate *P*. *xiamenensis* S1131.

### 2.2. Biochemical Characterization

The bacterium *P. xiamenensis* was positive for both oxidase and catalase activity, as it generated a blue color in dipped filters with 1% Koavacs oxidase reagent, and formed bubbles releasing oxygen due to the catalase enzymatic reaction, respectively. In contrast, the control *Escherichia coli* DH5α strain was catalase-positive and oxidase-negative. Further biochemical characterization was performed by Analytical Profile Index (API) 20E and 20NE assay platforms to analyze the enzymatic capabilities and fermentation of various sugars. As indicated in Supplementary Table S2, the *P. xiamenensis* isolate showed a positive readout on the API 20E assay platform only for the Voges−Proskauer reaction and gelatinase production, while for API 20NE it showed positive reactions for potassium nitrate and tryptophan fermentation, and could utilize aesculin, gelatin, arabinose, mannose, mannitol, N-acetyl-glucosamine, and gluconate. The reactions were compared against *E. coli* DH5α, which demonstrated distinct differences with the enzyme and sugar metabolism profiles. Thus, *P. xiamenensis’s* biochemical profiles indicate that it is non-Enterobacteriaceae (Figure S2). 

### 2.3. Antibiotic Sensitivity Profile

Antibiotic sensitivity was assayed against 12 selected antibiotics by disc diffusion assay. Size measurements of the inhibitory zone were taken (Table 1). The *P. xiamenensis* isolates were susceptible to chloramphenicol, ampicillin, ciprofloxacin, erythromycin, vancomycin, gentamycin, and imipenem, and were resistant to tetracycline, clindamycin, and penicillin. An intermediate level of resistance could be observed against the aminoglycoside antibiotics streptomycin and cefotaxime. 

### 2.4. Production and Secretion of Antimicrobials

An agar diffusion study was conducted to evaluate whether *P. xiamenensis* secretes antimicrobial compounds to the surrounding environment. Test bacterial strains *A*. *hydrophila, S*. *epidermidis, E*. *piscicida,* and *P*. *aeruginosa* (Figure S3) were spotted onto the soft overlay agar. After solidification, an agar plug was removed and filled with marine soft agar containing *P. xiamenensis*. Controls were selected with chloramphenicol (50 µg/mL) or marine agar medium alone. Culture plates were incubated at 28 °C for 24 h to develop a red-colored colony spread of *P. xiamenensis*. However, the strain did not create any antimicrobial diffusion area against the tested bacteria. Hence, the antimicrobial mechanism may be due to immune modulation or other mechanical antagonisms (Figure S3). 

### 2.5. In Vivo and In Vitro Safety Assessments 

As *P. xiamenensis* is a marine bacterium, we chose the zebrafish larvae model to evaluate its safety. Sixty hours post-fertilization (hpf), zebrafish larvae were exposed to variable doses of 7.88, 8.2, 8.4, and 8.5 log CFU/mL of *P. xiamenensis* in an egg water medium. At any inoculation dose between 7.6 × 10^7^ (log 7.88) and 1.6 × 10^8^ (log 8.2) CFU/mL conditions, no significant larval mortality was observed (complete survival). A 90% relative survival was observed at high inoculation doses at 2.6 × 10^8^ (log 8.4) and 3.8 × 10^8^ (log 8.5) CFU/mL; (Figure 2A,B). These observations were conducted over 96 h post-exposure (hpe). Importantly, no deformities, such as pericardial edema, spinal curvature, or head and tail malformations were observed during the test period. The heart rate of *P. xiamenensis-*exposed zebrafish larvae was 123.6 pulses/min, which was not significantly higher than the normal/untreated control. To further confirm the safety, an in vitro cytotoxicity assay was conducted using a fathead minnow (FHM) fish epithelial cell line. After inoculation at variable multiplicities of infection (MOI) of 10, 40, and 100, propidium iodide (PI) staining to stain late apoptotic or damaged cells was performed (Figure 2C). Compared to the pathogenic bacterium *E. piscicida,* at the same level of inoculation, *P. xiamenensis* did not cause significant cytotoxicity [16].

### 2.6. Augmentation of Disease Resistance

The effect of *P. xiamenensis* treatment on disease resistance was evaluated by pre-exposing zebrafish larvae to *P. xiamenesis* and subsequently challenging the fish with pathogenic bacterium *E. piscicida*. Larvae were co-cultured with *P. xiamenensis* in egg water for 180 h and then challenged with *E. piscicida*. At the end of 72 h post challenge (hpc), the *P. xiamenensis*-enriched group showed significantly higher (73.3%) survival compared to the *P. xiamenensis*-untreated, *E. piscicida-*treated larvae group (46.6% survival; Figure 3A). The naive control group demonstrated 90.0% survival at the end of the 72 h incubation period.

### 2.7. Augmentation of Heat Resistance

The effect of heat tolerance of zebrafish was investigated after *P. xiamenensis* exposure to the larvae. Zebrafish larvae were co-cultured with *P. xiamenensis* for 180 h and exposed to elevated temperature conditions (38 °C for 48 h). Interestingly, *P. xiamenensis* treated zebrafish larvae possessed a high level of heat resistance, demonstrating 95% survival after 24 h of exposure, compared with the non-treated group, which showed 85% survival. After 36 h of heat exposure, the *P. xiamenensis* treated larval survival was 90%, whereas for the non-treated group it was only 75%. At the end of the observation period, the *P. xiamenensis* treated larvae demonstrated 85% survival, whereas the non-treated larvae only showed less than 60% survival. No larval mortality was observed for the control group grown at normal culture temperatures (Figure 3B). 

### 2.8. Quantitative Real Time-PCR (qRT-PCR) Analysis 

**Figure 4:**
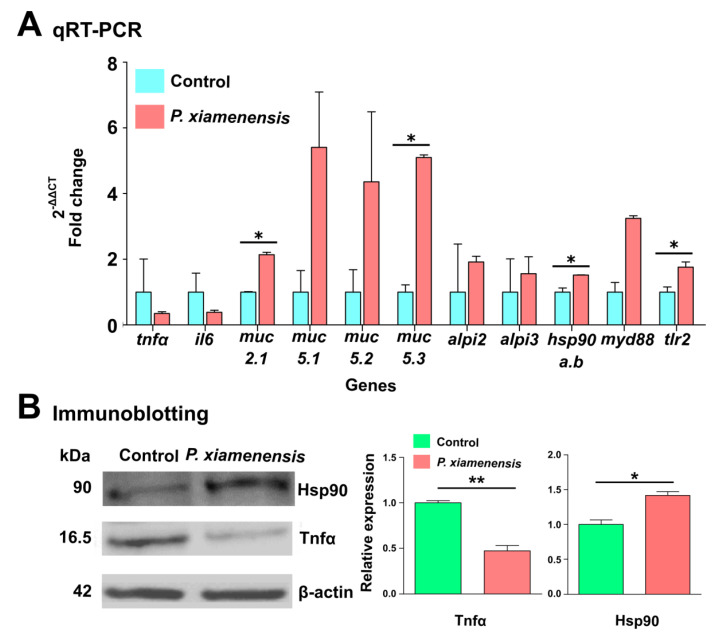
qRT-PCR and immunoblotting analysis of *P. xiamenensis*-treated zebrafish larvae. (**A**) The effect of *P. xiamenensis* treatment on immune-related genes at the mRNA level using qRT-PCR. Gene expression profiles were determined in non-treated control and *P. xiamenensis-*treated zebrafish treatment groups. Asterisk (*) indicates a significant difference compared to the non-treated control. Significance was set at *p* < 0.05. (**B**) Immunoblot analysis of Hsp90 and Tnfα in *P. xiamenensis-*treated zebrafish larvae, compared to the non-treated control. Expression levels of β-actin were set as the normalizing control. The intensity of each protein band was measured by Image J software. Asterisk (*) indicates a significant difference compared to the control group (* *p* < 0.05, ** *p* < 0.01). Statistical significance was set at *p* < 0.05.

The expression of the immunomodulatory genes of zebrafish larvae exposed to *P. xiamenensis* was evaluated at the mRNA level using qRT-PCR (Table S1). Genes selected for analysis were pattern recognition receptors (*tlr2*, *tlr4*, *tlr5a*, and *tlr5b*), signal transduction adaptor gene (*myd88*), pro- and anti-inflammatory cytokines (*tnfα*, *il6*, *il10*, and *il1β*), antimicrobial genes (*muc2.1, muc5.1*, *muc5.2*, *muc5.3*, *defbl1*, *defbl2*, *alp1*, *alpi2*, and *alpi3*), antioxidant enzymes (*sod1* and *catalase*), transcription factor (*nfkbia*), transforming growth factor (*tgfβ*), and stress-responsive and environmental adaptation-related genes (*hsp70*, *hsp90a.a*, and *hsp90.a.b*). Upon *P. xiamenensis* treatment, the *myd88* and *tlr2* genes were significantly upregulated by 3.25- and 1.76-fold, respectively, whereas the pro-inflammatory cytokine *tnfα* and *il6* genes were down-regulated by 2.86 and 2.63-fold, respectively (Figure 4A). Other *tlr* genes, *tlr4*, *tlr5a*, and *tlr5b*, were also down-regulated by *P. xiamenensis* treatment (Figure S4). Interestingly, the genes responsible for the synthesis of mucins were upregulated, including *muc2.1* (2.14-fold), *muc5.1* (5.41-fold), *muc5.2* (4.36-fold), and *muc5.3* (5.10-fold). Furthermore, *alpi2* and *alp3* were marginally upregulated at 1.92 and 1.56-fold, respectively. Moreover, the stress-responsive heat shock protein *hsp90.a.b.1* was upregulated by 1.52-fold in *P. xiamenensis* treated larvae, demonstrating its potential implications for environmental stress responsiveness [17].

### 2.9. Immunoblot Analsysis of Tnfα and Hsp90 Proteins

To further confirm the induction of heat-shock proteins and the suppression of pro-inflammatory cytokines, we conducted an immunoblot analysis. The total protein was extracted from zebrafish larvae pre-exposed to *P. xiamenensis* or non-exposed controls. There was a significant induction of Hsp90, and significant suppression of Tnfα proteins, by *P. xiamenensis* treatment (Figure 4B). Band intensity measurements revealed a Hsp90 and Tnfα relative expression at 1.42 (*p* < 0.05) and 0.47 (*p* < 0.01) for the *P. xiamenensis-*treated group. The β-actin gene was used as an internal control for the gene expression comparison.

### 2.10. Effect of Mucin on Colony Dispersion and Macrophage Uptake

**Figure 5:**
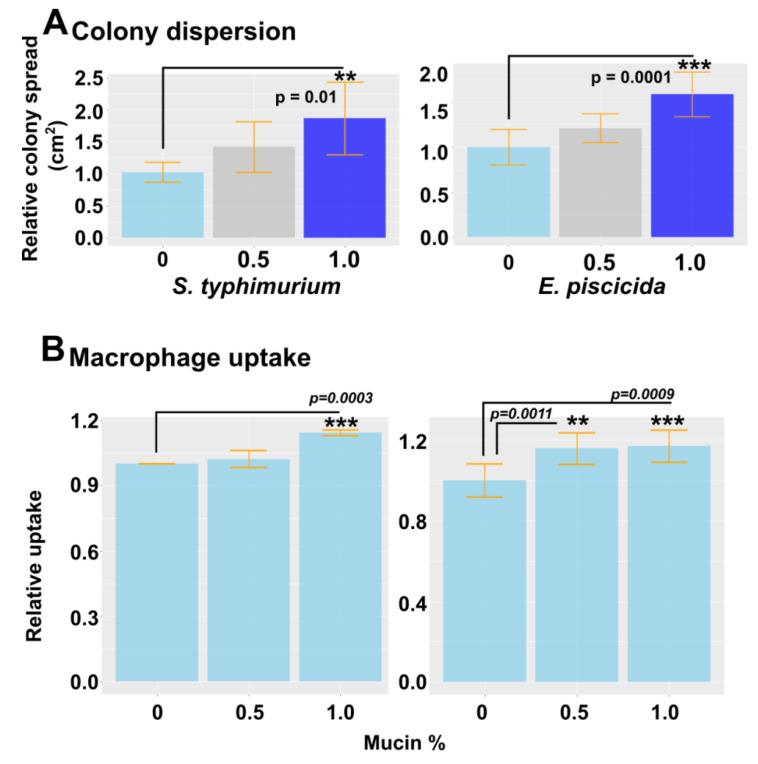
Mucin-dependent colony dispersion and macrophage uptake using motile pathogens *S*. *typhimurium* and *E. piscicida*. (**A**) The effect of mucin on colony dispersion/prevention of biofilms was investigated using the motile bacterium *S*. *typhimurium* and *E. piscicida*. Porcine mucin at variable concentrations was incorporated into soft agar and allowed to solidify. Mid-log phase bacterial culture was spotted on the agar. After 12 h incubation at 28 °C for *E. piscicida* and 37 °C for *S. typhimurium*, the colony diameter was measured. ** and *** indicate a significant difference compared to the non-treated control. (**B**) The mouse macrophage RAW264.7 cell line was seeded in 24-well plates at 5 × 10^4^ cells/well. Cell media was supplemented with porcine mucin at variable concentrations. *S. typhimurium* and *E. piscicida* were inoculated at MOI 40 and 100. After 1 h of co-incubation at 37 °C, the cells were washed with PBS three times, treated with gentamycin, and lysed with 0.05% Triton X-100 PBS. The whole lysate was collected and serially diluted for plating on agar. Internalized bacterial numbers were determined by CFU counting. The relative uptake was calculated based on the uptake of the control. ** and *** indicate a significant difference compared to the non-treated control. The level of significance was set at *p* < 0.05.

Mucin is a frontline defense barrier in the innate immune system. Due to significant changes in the mucin gene expression induced by *P. xiamenensis* treatment, we hypothesized that there could be a potential link between this and pathogen resistance. To assess the effect of mucin on bacterial colony formation, we used motile bacteria *E*. *piscicida* and *Salmonella enterica* serovar *typhimurium* (Figure 5). Commerciallyavailable porcine mucin was added to a 0.8% soft agar medium at variable concentrations (0, 0.5, or 1.0%). Mid-log phase *E. pscicida* and *S. typhimurium* were dotted on solidified agar and incubated overnight. The next day, the colony diameters were measured, and we observed the dispersion of colony size, possibly due to the prevention of tight biofilm formation. This demonstrated the mucin’s ability to dissolve biofilms as a defense strategy against pathogenic bacteria. With an increase in mucin concentration, a higher level of colony dispersion was observed, resulting in larger colonies (Figure 5A). The effect of mucin on macrophage uptake was also investigated by incorporating porcine mucin into the cell culture media in which murine macrophage RAW264.7 cells were grown. Cell medium was supplemented with 0, 0.5, or 1.0% porcine mucin, and the *E. piscicida* and *S*. *typhimurium* bacteria were inoculated at a multiplicity of infection (MOI) of 40. After 1 h of incubation, the cells were washed with gentamycin to eliminate bacteria in the surrounding media, and the number of bacteria taken up by macrophage cells was quantified by plating on agar with serial dilutions. A significant increase in macrophage uptake was evident when 1.0% mucin was added to the agar, suggesting that mucin may increase macrophage uptake, and may potentially enhance antigen presentation against invasive bacteria (Figure 5B).

## 3. Discussion

Marine bacteria belonging to the genus *Pseudoalteromonas* have been known to produce various antibacterial and antiviral compounds, and thus may have potential for various health benefits for the host [18]. However, identification of novel bacterial candidates is a difficult and time-consuming procedure that requires extensive characterization of the safety aspects and potential benefits [19]. It also must contain several desirable features for it to be considered as a candidate probiotic strain [20]. Probiotics of a marine origin could represent a sizeable demand for future human consumption; thus, their applications are worth exploring. This study describes the isolation and characterization of a newly-isolated *Pseudoalteromonas* species, and its healthful, beneficial effects on the zebrafish model determined with a focus on disease and environmental tolerance. 

The bacterium was originally isolated from marine water collected from Chuuk state, Federated States of Micronesia. Hence, the bacterium ideally should be investigated in a fish model. After enrichment and sequential colony purification, the strain was subjected to further characterization. On streaked plates, the colonies appeared to be convex in shape, watery in texture, and red in color at late incubation time points. The bacterium was a Gram-negative rod-shaped bacterium. Under a scanning electron microscope, it appeared to secrete a thick extracellular matrix, which could retain antimicrobial properties, exerting an antagonistic effect on pathogenic bacteria. Biochemical characterization of the isolate by API 20E and 20NE assay platforms revealed that the current *P. xiamenensis* isolate was negative for most of the reactions on the 20E assay platform (Figure S2), which was quite different from the *Enterobacter* species *E. coli*. Interestingly, the present bacterial isolate did not show a positive reaction for glucose acidification, and was positive for the Voges−Proskauer reaction, as opposed to some reported species of *P. xiamenensis* [21]. Biochemical profiling alone is not sufficient for the species-level prediction of unknown species. Hence, we conducted 16S rRNA sequencing for precise molecular level identification. A EzBioCloud 16S rRNA database search revealed the isolated *Pseudoalteromonas* species to be an isolate of *P. xiamenensis* belonging to the class Gammaproteobacteria (Supplementary Figure S1) [22]. The bacterium could grow at temperatures between 15–40 °C, with optimal growth at tropical temperatures (35 °C). Acidic conditions below pH 5 were lethal to bacterial growth; however, it demonstrated significant tolerance at alkaline pH values up to pH 10. Peak growth was reached at neutral pH (pH 7). Therefore, the majority of the orally engulfed bacteria may survive in the intestinal environments of fish where the pH range does not normally reach below 7.5 under homeostatic conditions [23].

The safety of *P. xiamenensis* was of paramount importance for its use as a probiotic strain. Co-exposure experiments after inoculating into the water containing zebrafish larvae revealed no induction of mortality even at the highest inoculation doses (8.5 log CFU/mL). No abnormality of feed intake, behavior, or malformation of body configurations was observed; thus, it could be defined as a non-pathogenic organism. Furthermore, compared to a pathogenic *E. piscicida* strain, the present *P. xiamenensis* strain did not cause significant cell death in in vitro experiments (Figure 2). The *P. xiamenensis* isolate is also unlikely to produce harmful substances such as H_2_S, as indicated by a biochemical test. Thus, our isolate should be safe to utilize as a probiotic strain [24]. Antibiotic sensitivity profiling also demonstrated susceptibility to most tested antibiotics, including chloramphenicol, ampicillin, ciprofloxacin, erythromycin, vancomycin, gentamycin, and imipenem, and partial susceptibility to streptomycin and cefotaxime. Thus, in the case of an emergency pathogenic infection, it can be reliably controlled by the application of a suitable antibiotic regimen. 

Disease tolerance induced by probiotic strains is governed by multiple mechanisms. Immune modulation, competitive inhibition, and competition for substrates are some of the proposed mechanisms [25]. Here too we observed that exposure of *P. xiamenensis* to zebrafish larvae confers significant tolerance against a subsequent lethal *E. pisicida* challenge. Pathogenic infection with *E. piscicida* is capable of killing infected larvae with high mortality rates in just two days. However, the survival of *P. xiamenensis-*treated fish for more than three days suggests that there is an augmentation of disease tolerance from the probiotic treatment. In diffusion experiments, we observed that *P. xiamenensis* itself does not secrete any antimicrobial substance inhibiting the tested bacterial strains. Hence, it can be speculated that the observed enhancement of zebrafish larvae resistance may be largely due to host immune stimulation. In addition, treatment with *P. xiamenensis* improved temperature tolerance, as demonstrated by heat shock experiments conducted at 38 °C; *P. xiamenensis-*treated fish larvae were capable of surviving at a high temperature. These larvae are highly sensitive to temperature shifts; thus, their adaptation to elevated temperatures is a clear indication of the effect of probiotic treatment. The underlying reason might be related to the mucin-related activity in the host, as mucin signaling cascades are known to regulate thermal stress adaptations [26]. Most probiotic strains are Gram-positive, such as *Lactobacillus bifidobacteria* [27]. Gram-negative bacterial strains have also been utilized as probiotics, for example *E. coli* Nissle 1917, used to treat chronic constipation and colitis in Germany [28]. As immune modulators, Gram-negative probiotic strains may be superior to that of Gram-positive strains, as the outer membranes of Gram-negative bacteria are antigenically more complex and are potent inducers of host immunity. To understand the immune-modulatory landscape, we conducted a qRT-PCR assessment of the marker genes, which revealed that *P. xiamenensis-*treated larvae significantly upregulated mucin-related genes and suppressed pro-inflammatory markers. Treatment with *P. xiamenensis* also induced heat-shock protein 90 expression. Marginal induction of the *tlr2* and MyD88 signaling molecules may induce antimicrobial defense in zebrafish. Noticeably, there was no significant engagement of other TLR receptors that are associated with inflammation (Supplementary Figure S1), such as *tlr4*, *tlr5a*, and *tlr5b*. These immunological differences indirectly demonstrate noncanonical lipopolysaccharides that do not cause significant endotoxicity [29]. The low level of inflammation can be a favorable feature of the present *P. xiamenensis* isolate for it to be utilized as a probiotic strain. In this context, the disease tolerance might at least partially be attributed to the induction of mucin secretion as one of the first-line defense strategies associated with the innate immune system. Mucin creates a thick matrix with a highly glycosylated protein that contains antimicrobial compounds and acts as a physical barrier against bacterial penetration [30]. It also traps microbes, prevents biofilm formation, and enhances antigen presentation by professional antigen-presenting cells resident in the mucosal layers. This also promotes macrophage uptake and leads to rapid elimination of pathogens, without permitting the colonization of pathogens [31] on mucosal surfaces [32]. The effect of mucin could be demonstrated by the motile bacteria *E. piscicida* and *S. typhimurium* strains, which showed that mucin can prevent thick biofilm formation, cause dissolution of biofilms, and promote uptake by macrophages. However, the effect of mucin macrophages is connected with more complicated signaling networks, where the effect of mucin can be dependent on the phenotype of macrophages, whether they are M1, M2, or M0 [33,34]. Some reports have also demonstrated that certain pathogens, such as *P. aeruginosa*, utilize monosaccharides present in mucin to promote their infection [35]. Interestingly, *P. xiamenensis* treatment promoted heat tolerance of zebrafish larvae, which may be correlated with an increased expression of heat-shock-related stress proteins such as Hsp90. These constitutive genes not only promote heat resistance, but also resistance against salt stress [36,37]. Importantly, marine bacterial species possess more stable enzymatic pathways and a higher level of tolerance against environmental changes than other bacterial species. Thus, they could be better suited as probiotic strains than bacterial species of an intestinal origin [38]. These enzymes are also functionally active at a relatively wide range of physicochemical conditions, and can also promote host functions such as digestion.

In summary, we propose that marine bacterial species, such as *P. xiamenenssis*, can be an enormous potential as probiotic species, which are safe and environmentally friendly, yet could significantly augment host defense against diseases and environmental assaults. We also demonstrated zebrafish as an appropriate animal model to validate marine-isolated bacteria, whose applications can be easily adapted from other acclaimed animal models. The underlying mucin-derived anti-inflammatory properties of marine-isolated bacteria, such as *P. xiamenensis*, may redefine future perspectives on probiotic bacteria. We encourage the search for more candidates, to cater to industries such as the aquatic animal, food, and health industries.

## 4. Materials and Methods

### 4.1. Identification and Characterization

#### 4.1.1. Isolation and Morphological Characterization 

Strain S1131 was originally isolated from a seawater sample collected from Chuuk state, Federated States of Micronesia. The strain was streaked on marine agar (BD, Sparks, MD, USA) and grown at 30 °C. The colony characteristics including the form, color, size, elevation, and margins of the purified bacterial colonies were evaluated by visual and microscopic observation. The Gram status was determined by the standard Gram staining procedure [39]. Microscopic analysis of the bacterium was conducted by scanning electron microscopy. Briefly, cells were washed with phosphate-buffered saline (PBS) to remove media contaminations and were mounted on slides for air drying. Cells were coated with conductive material (chromium), and surface scanning was done by the Supra 40VP Gemini Scanning Electron microscopic system (Zeiss, Oberkochen, Germany) at a 10 kV acceleration voltage. Cell dimensions were measured using ImageJ Fiji software on at least 20 individual colonies or cells [40]. Purified bacterial single colony isolates were prepared and stored in 40% glycerol at −80 °C until future use.

#### 4.1.2. Phylogenetic Analysis

The 16S rRNA sequence of strain S1131 was amplified from a pure colony using the universal bacterial primers 27F and 1492R, from Macrogen Inc. (Seoul, South Korea). The sequence was determined, and the closest phylogenetic neighbors were then identified using the EzTaxon-e database (https://www.ezbiocloud.net/identify, accessed on 9 December 2021). Phylogenetic analysis was conducted with type strains of *Pseudolateromonas* species. Alignments were carried out using the CLUSTAL × 2.1 program [31], with the gaps edited using the BioEdit program [41]. A distance matrix was produced on the basis of Kimura’s two-parameter model [42]. A phylogenetic tree was generated by the neighbor-joining [43] method in the MEGA 7.0 program [44]. The topologies of the resultant trees were computed in a bootstrap analysis of 1000 replicates.

#### 4.1.3. Biochemical Characterization 

The *P. xiamenensis* isolate was evaluated for oxidase and catalase activities, and subjected to API 20E/NE tests. The oxidase and catalase tests were performed following the standard protocols described in previous publications [45,46,47]. The oxidase activity of *P. xiamenensis* was determined by picking a well-isolated colony from an 18–24 h incubated culture plate and was streaked on a filter paper presoaked with 1% Kovacs oxidase reagent. The chromogenic changes were observed after 5 min of incubation at room temperature (25 °C). To test for the catalase activity, a 3% hydrogen peroxide (H_2_O_2_) drop was placed on a dry clean glass slide, and a loop of bacteria was then spread on the H_2_O_2_. The rapid evolution of air bubbles from H_2_O_2_ by the catalase enzyme activity was evaluated. Biochemical characterization was conducted using bioMerieux’s API 20E and 20NE assay platforms according to the manufacturer’s recommendations (Bio-Mérieux, Basingstoke, Great Britain). Briefly, the bacterial inoculums were prepared using overnight grown cultures of bacteria on marine agar. The inoculums were prepared in 4 mL of marine broth and inoculated into API 0.85% NaCl medium. Then, the inoculums were inoculated into 20E and 20NE test strips. The cultures were incubated at 30 °C for 15 h in a shaking incubator at 180 rpm. The test strips inoculated with bacteria were observed after 24 to 48 h using the reference guide provided. 

#### 4.1.4. Determination of Optimum Temperature and pH

The temperature susceptibility of the isolate was conducted by determining the growth at various physiological temperatures. Briefly, 190 μL of marine broth was inoculated with 10 μL of an overnight culture of bacteria that contained 3.8 × 10^8^ CFU/mL in 96-well plates. Bacterial suspensions were incubated at 15, 20, 25, 30, and 35 °C. The growth of the bacteria was measured at 0, 4, 8, 12, 20, and 24 h by determining the absorbance (MarkTM, Bio-Rad Saint Louis, MO, USA) at 595 nm (OD_595_) after thoroughly mixing the culture. The absorbance at the 24 h time point was compared and presented. The pH-dependent growth tolerance was tested by culturing the *P. xiamenensis* in marine broth at a pH ranging from 3 to 10 at 30 °C for 24 h in a shaker (180 rpm). Bacterial growth was determined by measuring the OD_595_ at 0, 4, 8, 12, 20, and 24 h. The absorbance at the 24 h time point was compared.

### 4.2. Analysis of Antibiotic Sensitivity

The antibiotic susceptibility of the *P. xiamenensis* isolate was conducted against chloramphenicol, ampicillin, ciprofloxacin, erythromycin, gentamycin, imipenem, streptomycin, tetracycline, cefotaxime, clindamycin, and penicillin. The concentrations of each antibiotic are listed in Table 1. The bacterial lawns were prepared by overlaying bacterial-inoculated soft agar (0.8%). Briefly, 4 mL of soft agar was prepared using marine agar and heated in a water bath. When the agar was fully dissolved, the media containing test tubes were allowed to cool down for bacteria inoculation. One hundred microliters of overnight broth were mixed into soft agar and overlaid onto marine agar plates. The cultures were allowed to solidify at room temperature. Antibiotic-soaked discs were aseptically placed over the bacterial lawns and incubated at 30 °C for 24 h. The antibiotic susceptibility was determined by the presence or absence of inhibition zones, and the zone dimensions were measured for comparison [48].

### 4.3. Secretion of Antimicrobials

Secretion of antimicrobials by *P. xiamenensis* was assessed by diffusion assay on agar. Soft agar (0.8%) was prepared using Brain Heart Infusion (BHI) broth (BD, Sparks, MD, USA) or Tryptic Soy Broth (TSB, BD). The bacterial strains *E. piscicida, S. epidermidis*, *A. hydrophila*, and *P. aeruginosa* were incorporated into 4 mL of soft agar and overlaid onto pre-solidified respective agar media. *P. xiamenensis* was grown either in marine agar or broth. *E. piscicida* was grown on BHI broth medium while other strains were propagated on Tryptic Soy Broth (TSB; BD, Reno-Sparks, NV, USA) medium. Briefly, overnight cultures were inoculated into fresh respective mediums, 1% *v*/*v*, and allowed to grow to 0.4 OD_595_. At the appropriate density, 200 µL was retrieved and suspended into 4 mL of soft agar prepared by the respective media for each strain. The suspensions were overlaid onto pre-solidified agar and allowed to solidify. Then, complete round plugs were removed and filled with *P. xiamenensis* suspended in marine agar in a similar procedure. The *P. xiamenensis* suspension (1 mL) was poured into the well and allowed to solidify. Marine soft agar alone, or chloramphenicol-treated (50 µg/mL), were used as negative and positive controls, respectively. Plates were incubated> 24 h for pink color development, and the diffusion zones were compared. 

### 4.4. Dose Optimization and Safety

The *P. xiamenensis* overnight-grown culture was inoculated into 10 mL of fresh marine broth at 1% (*v*/*v*) inoculation. Cultures were incubated at 30 °C with vigorous shaking. When the OD_595_ reached 0.4 (1.6 × 10^8^ CFU/mL), the cells were collected by centrifugation at 2657× *g* at 4 °C for 10 min. The supernatant was discarded and the pellet was collected and re-suspended in 1 mL of PBS and placed on ice. Cell numbers were adjusted to 0.2, 0.4, 0.6, and 0.8 OD_595_, to bring cell numbers to 7.6 × 10^7^, 1.6 × 10^8^, 2.6 × 10^8^, and 3.8 × 10^8^ CFU/mL, respectively. Zebrafish at 60 hpf were collected into 6-well plates containing egg water, and co-inoculated with *P. xiamenensis* by adding 200 μL of each inoculation dose in replicates. Fish larvae without bacterial inoculations were test controls. Plates containing fish larvae and bacteria were incubated at 28 °C. Larval mortality was monitored for 96 h. The morphological changes were observed under an optical microscope (Leica Biosystems, Wetzlar, Germany). The average heart rate was also evaluated for the control and treatment groups under microscopic observation. 

### 4.5. In Vitro Cytotoxicity Assay

The safety of *P. xiamenensis* was evaluated by in vitro cytotoxicity induction using FHM cells. Cells were grown in 24-well plates in complete medium, Dulbecco’s Modified Eagle’s’ medium (DMEM; Lonza, Basel, Switzerland) plus 10% Fetal Bovine Serum (FBS). When the cells reached >70% confluence, they were treated with *P. xiamenensis* and *E. piscicida* at MOIs of 10, 40, and 100. After 24 h of incubation in a humidified 5% CO_2_ incubator, the cells were fixed with 4% paraformaldehyde solution for 20 min at room temperature. The cells were washed with PBS two times and stained with PI (2 µg/mL). The treated cells were further incubated for 30 min on ice in the dark and visualized by fluorescent microscopy (Leica, Wetzlar, Germany).

### 4.6. Disease-Resistance Capacity of P. xiamenensis-Enriched Zebrafish Larvae

The development of disease resistance by *P. xiamenensis* enrichment was investigated using zebrafish larvae challenged with *E. piscicida*. Zebrafish larvae collected at 0–1 hpf were incubated at 28 °C, with a 12/12 h light and dark cycle in sterile egg water. At 60 hpf, the hatched larvae were transferred into a new plate containing egg water at 50 larvae per 50 mL of egg water in a petri dish. The plates were then incubated at 28 °C for 3 h. Treatment by larval enrichment was conducted by inoculating with 3.8 × 10^8^ CFU/mL *P. xiamenensis* per petri dish and continued incubation at 28 °C. The second and third enrichments were done at 6 days post-fertilization (dpf) and 9 dpf. Feeding of larvae with egg yolk and *Artemia* was initiated at 6 dpf and was continued throughout the experiment period. The control group was not supplemented with *P. xiamenensis*. All experiments were conducted in three independent trials in replicates. After the treatment period, a challenge was conducted at 10 dpf with *E. piscicida* at an inoculation dose of 1.35 × 10^9^ CFU/mL. Briefly, zebrafish larvae were subjected to heat stress at 34 °C for 2 h. The challenge strain *E. piscicida* was inoculated into 4 mL of BHI medium containing 1% NaCl and was incubated overnight at 25 °C with vigorous shaking (180 rpm). When the OD_595_ reached 0.4, the cultures were centrifuged at 2657× *g* for 10 min at 4 °C. The pellet was re-suspended in 1 mL of PBS and kept on an ice bath. The OD_595_ values were adjusted to receive the inoculation dose. After the challenge, plates were incubated at 28 °C. Larval mortality was recorded in 12 h intervals for 72 hpc. 

### 4.7. Heat Resistance Capacity of P. xiamenensis-Enriched Zebrafish Larvae

To assess the heat tolerance of *P. xiamenensis* treated zebrafish larvae, the raising of fish larvae and treatments were carried out similarly as for the disease resistance. At 10 dpf, zebrafish larvae that had been treated with *P. xiamenensis* (*P. xiamenensis* only or *P. xiamenensis* and heat stress) and non-treated groups (control) were exposed to heat stress at 38 °C. For each group, 10 larvae were utilized in 10 mL egg water in six-well-plates. The mortality was monitored for 60 h in 6 h intervals. 

### 4.8. Transcriptional Analysis of Immunomodulatory Genes

Expression of immunomodulatory genes in *P. xiamenensis*-treated zebrafish larvae were evaluated by qRT-PCR. The treatment of zebrafish larvae with *P. xiamenensis* was initiated at 60 hpf and continued until 10 dpf, as described earlier. At 10 dpf, 30 larvae per replicate were collected and frozen in liquid nitrogen and stored at −80 °C until RNA isolation. The total RNA isolation from the whole larval body was conducted using Trizol reagent (Sigma-Aldrich, St. Louis, MO, USA), according to the manufacturer’s instructions. The RNA concentration of each sample was measured using NanoDrop One (Thermo Scientific, Waltham, MA, USA) and a total of 2.5 µg of total RNA was reverse-transcribed by the Prime Script™ first-strand cDNA synthesis kit (TaKaRa^®^, Tokyo, Japan). Synthesized cDNA samples were further diluted and qRT-PCR was performed to analyze the expression of selected immunomodulatory genes in *P*. *xiamenensis*-enriched zebrafish larvae and controls using a Thermal Cycler Dice Real-Time System (TaKaRa^®^, Tokyo, Japan). The list of primers used is shown in Supplementary data Table S1. Specific conditions used for thermal cycling are mentioned in a previous publication [49]. Zebrafish β-actin was used as a housekeeping gene. The expression fold was calculated using the 2^−(∆∆CT)^ method [50].

### 4.9. Immunoblot Analysis

The immunoblot analysis of Tnfα and Hsp90 was done using whole-body protein extracts collected from 10 dpf zebrafish larvae. Briefly, *P. xiamenensis-*treated and non-treated larvae (30 individuals per treatment) were collected and homogenized in 200 μL of ice-cold lysis buffer, pH 7.6 (ProEXTM CETi, TransLab Inc., Daejeon, Korea). Homogenized samples were centrifuged at 9695 g for 10 min at 4 °C. Quantification of the proteins was conducted using the Bradford assay [51] (Bio-Rad Laboratories, Hercules, CA, USA). Protein extracts were mixed with an equal volume of 2 × SDS-PAGE sampling buffer (Sigma, St. Louise, MO, USA) and denatured by boiling at 95 °C for 5 min. Thirty-five micrograms of each sample were loaded into a 12% SDS-PAGE gel and electrophoresed. The stacking voltage was set at 80 V for 30 min and the running voltage was set at 110 V for 2 h. 

The resolved proteins were then transferred onto a PVDF membrane and blocked with 5% bovine serum albumin (BSA, GeorgiaChem, Seoul, Korea) for 2 h. After blocking, the membranes were washed three times with 0.01% PBST and incubated with Tnfα (KP1540Z-100; Kingfisher Biotech, Saint Paul, MN, USA) or Hsp90 (4874S; Cell Signalling Technology, Danvers, MA, USA) antibodies at 1:1000 dilutions, or β-actin specific monoclonal antibodies (sc47778; Santa Cruz Biotechnology, Dallas, TX, USA) at 1:3000 dilution overnight at 4 °C. The membranes were washed with PBST four times and incubated with HRP-labeled secondary antibodies at 1:3000 dilution (Anti-mouse IgG (gtx213111-01; GeneTex, Irvine, CA, USA) or anti-rabbit IgG (7074 s; Cell Signaling Technology)) in 5% BSA at 25 °C for 2 h. Membranes were treated with 3,3-Diaminobenzindine (Sigmafast, St. Louise, MO, USA). Detection of specific proteins was carried out using a chemiluminescence detection system (Fusion Solo S, Vilber, Lourmat, France). The protein band intensities were quantified using the Evolution-CAPT software (FUSION software user and service manual-v17.03, Vilber Company, San Sebastiàn, Spain), and normalized against the expression of β-actin. Three independent experiments were carried out to quantify the average expression of Tnfα and Hsp90.

### 4.10. Effect of Mucin on Bacterial Dispersion and Biofilm Inhibition

The effect of mucin on bacteria biofilm formation inhibition and dispersion was evaluated by dotting assay on mucin-supplemented soft agar using fish pathogens *S. typhimurium* and *E. piscicida*. The porcine mucin (Sigma) was incorporated into soft agar at 0, 0.5, or 1.0% final concentrations. Soft agar was overlayed on normal Luria Bertani (LB; BD, Sparks, MD, USA) agar and allowed to solidify. *S. typhimurium* and *E. piscicida* were grown in 37 °C and 28 °C, respectively. Mid-log phase culures of *S. typhimurium* and *E. piscicida,* (motile pathogens,) were spotted onto solidified soft agar. Plates were incubated for 16 h at 37 °C for *S. typhimurium* and 28 °C for *E. piscicida*. The diameters of colony spread were measured and compared against the control plates that did not contain mucin supplements. 

### 4.11. Effect of Mucin on Macrophage Uptake

The effects of mucin on bacterial uptake by macrophages were evaluated on the RAW264.7 cell line. RAW cells were seeded in 24-well plates at a seeding density of 5 × 10 cells/well. When the cells reached 70% confluence, the cell media was removed and replaced with complete media containing 0, 0.5, or 1.0% mucin. *S. typhimurium* and *E. piscicida* were grown in their ideal growth temperatures at 30 °C and 37 °C, respectively, using appropriate media—LB for *S. typhimurium* and BHI for *E. piscicida*. When the cells reached mid log phase, they were collected and adjusted to MOI 40 and 100, respectively, and incubated for 1h at 37 °C, allowing for uptake. Wells were washed with PBS three times and replaced with complete medium containing 100 µg/mL gentamycin for 2 h. After incubation, the cell monolayers were lysed by adding 1 mL of PBS containing 0.5% Triton X100 (Sigma, St. Louis, MO, USA), and the whole lysate was collected. One hundred microliters of lysate were plated onto LB or BHI agar in dilutions for colony counting. 

### 4.12. Statistical Analysis

All the statistical data were analyzed using GraphPad Prism software version 5 (GraphPad Software Inc., La Jolla, CA, USA), and one-way analysis of variance (ANOVA) and/or unpaired *t*-test were performed to determine the statistical significance (* *p* < 0.05, ** *p* < 0.01, or *** *p* < 0.001). The in-vivo survival data were analyzed using a Log-rank (Mantel−Cox) test to identify the significance (*p* < 0.05) between the control and *P. xiamenensis* treatment. The data are shown as the mean ± standard error (SE) of triplicate experiments.

## Figures and Tables

**Table 1:** Antibiotic sensitivity profile of *P. xiamenensis* S1131.

Antibiotic	Tested Concentration (µg/disc)	Diameter of Zone/mm	Reference			Status
			Resistant (< or = mm)	Intermediate (mm)	Susceptible (= or > mm)	
Chloramphenicol	30	28.0	12	13–17	18	Susceptible
Ampicillin	10	15.0	11	12–13	14	Susceptible
Ciprofloxacin	5	22.0	15	16–20	21	Susceptible
Erythromycin	15	27.0	13	14–22	23	Susceptible
Vancomycin	30	15.0	9	10–11	12	Susceptible
Gentamycin	10	18.0	12	13–14	15	Susceptible
Imipenem	10	25.0	19	20–22	23	Susceptible
Streptomycin	10	17.0	14	15–20	21	Intermediate
Cefotaxime	30	25.0	8	16–31	32	Intermediate
Tetracycline	30	9.0	14	15–18	19	Resistant
Clindamycin	2	0.0	14	15–20	21	Resistant
Penicillin	10	0.0	14		15	Resistant

## Data Availability

The data presented in this study are available on request from the corresponding author.

## Supplementary Materials

The following are available online at https://www.mdpi.com/article/10.3390/md19120707/s1. Figure S1: Phylogenetic classification of *P. xiamenensis* isolate. Figure S2: Biochemical characterization of *P. xiamenensis*. Figure S3: Assessment of antimicrobials secretion of *Pseudoalteromonas xiamenensis*. Figure S4: Quantitative real-time PCR for additional cytokines. Table S1: Bacterial strains and primers used in the study. Table S2: Analytical Profile Index (API) 20E and 20NE biochemical test results of *P. xiamenensis*.
