# Supplementary material for: A Novel Pseudoalteromonas xiamenensis Marine Isolate as a Potential Probiotic: Anti-Inflammatory and Innate Immune Modulatory Effects against Thermal and Pathogenic Stresses
Source: Mar Drugs. 2021 Dec 15;19(12):707. doi: 10.3390/md19120707 (PMC8707914; doi:10.3390/md19120707)
Supplement: Supplementary file 1 [file marinedrugs-19-00707-s001.zip › marinedrugs-1459324-supplementary.pdf]

Supplementary Materials

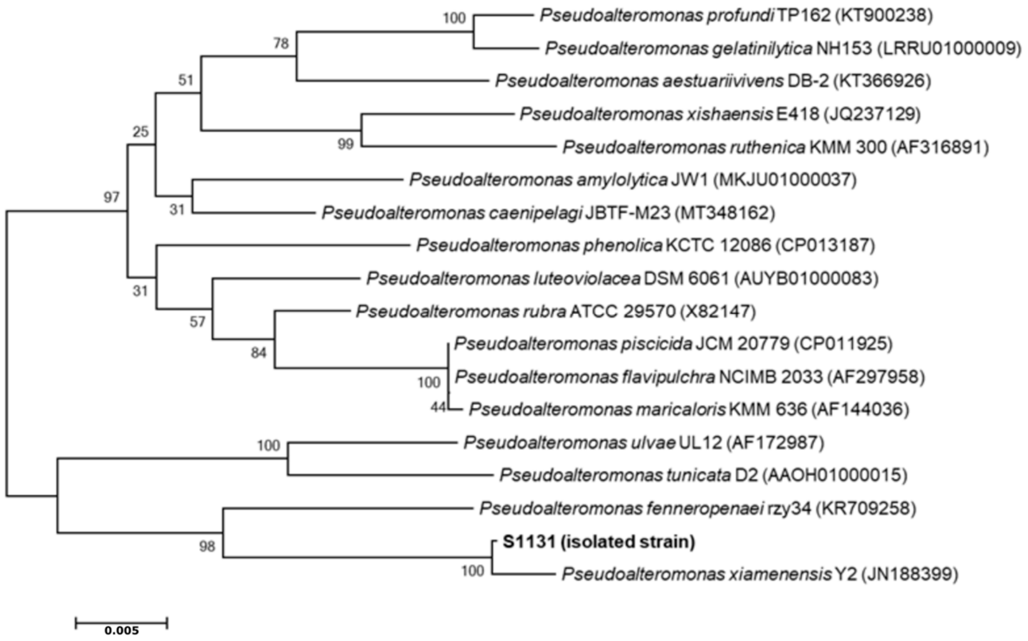

Figure S1. Phylogenetic tree based on 16S rRNA sequences showing the relationship of strain S1131 with type strains of the genus *Pseudoalteromonas*. Accession numbers are given in parenthesis. Bar, 0.005 substitutions per nucleotide position.

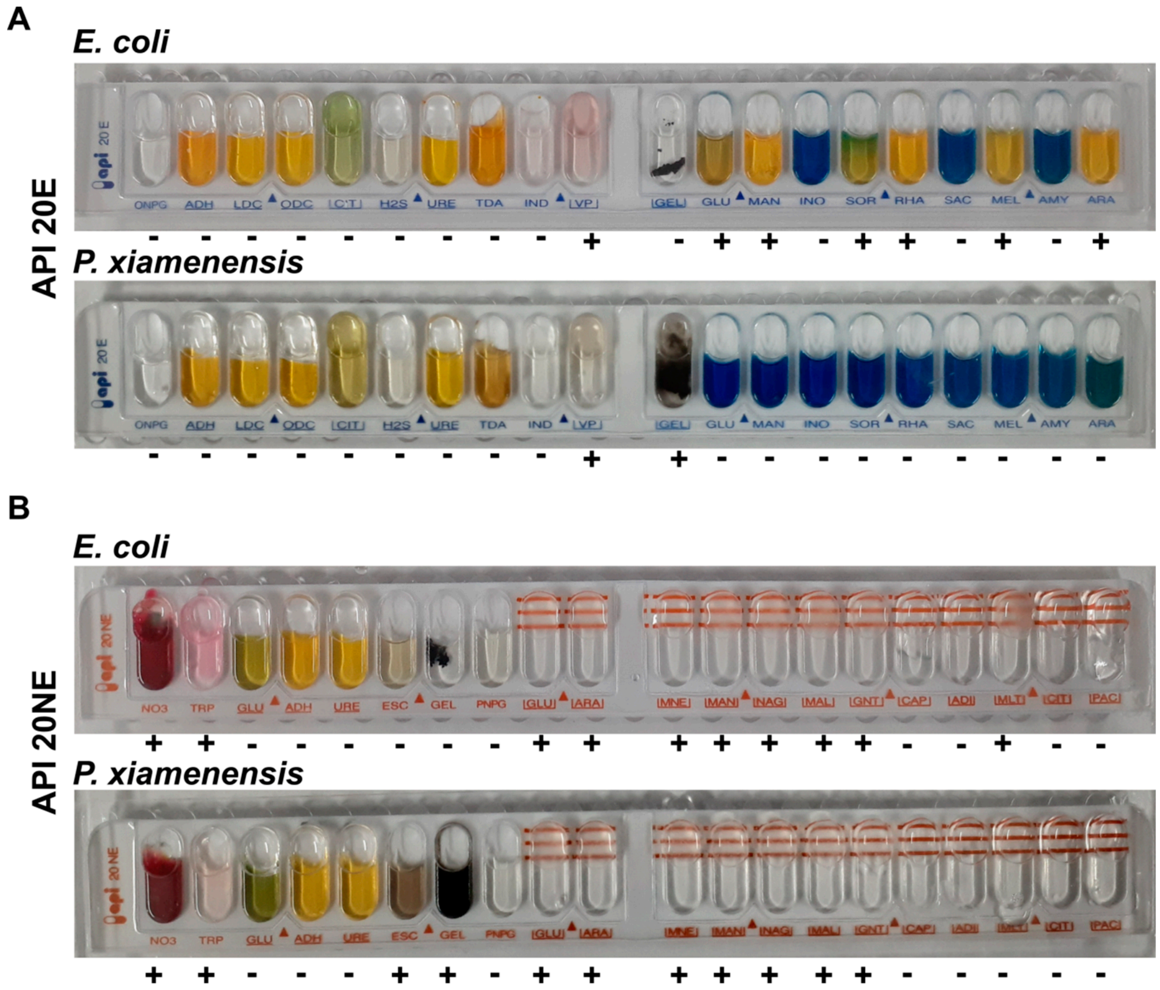

Figure S2. Biochemical characterization of *P. xiamenensis*. (A) API 20E assay results of *P. xiamenensis* as compared to *E. coli* DH5α. (B) API20NE assay results of *P. xiamenensis* as compared to *E. coli* DH5α. + mark indicates positive reaction and – mark indicates the negative reaction.

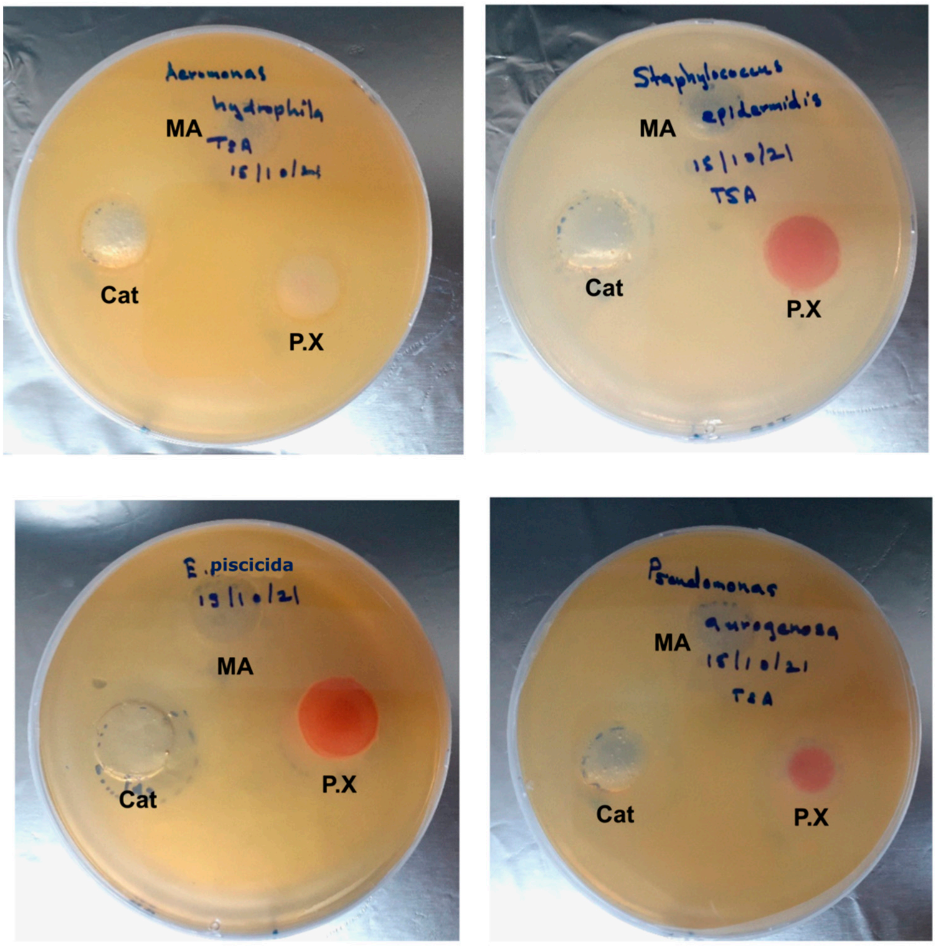

MA: Marine agar (0.8% soft agar)

Cat : Chloramphenicol 50 µg/mL

P.X: *P. xiamenensis*

Figure S3. Assessment of antimicrobials secretion of *P. xiamenensis*. Agar diffusion test was conducted to assess the inhibition ability of *P. xiamenensis* on *A. hydrophila, S. epidermidis, E. piscicida,* and *P. aeruginosa.* The test bacterium was inoculated into the soft agar and overlayed on the pre-solidified agar plates. Wells were cut and filled with marine agar containing *P. xiamenensis*. MA: Marine agar (0.8 % soft agar), Chl: chloramphenicol (50 µg/mL), P.X: *P. xiamenensis*.

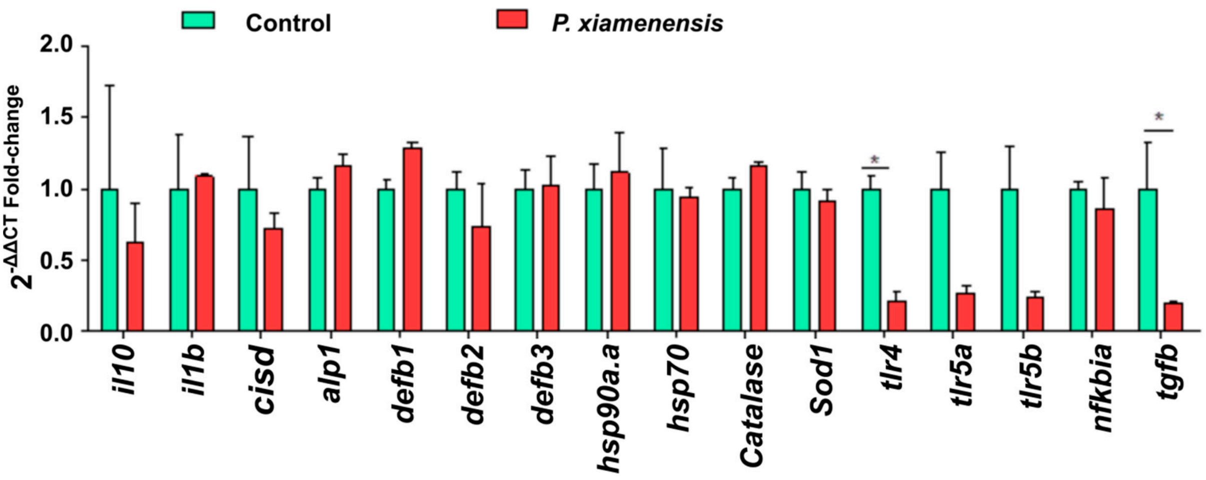

Figure S4. Quantitative real-time PCR for cytokines. Relative mRNA expression of immune-modulatory genes in *P. xiamenensis* enriched zebrafish larvae. Zebrafish larvae (60 hpf) were enriched with *P. xiamenensis* three times at 60 hpf, 6 dpf, and 9 dpf and were sampled at 10 dpf for isolating the RNA. Relative expression-fold is presented as mean ± standard error. The asterisk marks are used to indicate statistical significance (t-test *, $p < 0.05$).

Table S1: Bacterial strains and primers used in the study

Bacterial strain/Gene name	Reference/Accession number	Primer name	Primer sequence 5′-3′
**Bacteria strains**			
Pseudoalteromonas xiamenensis S1131	Gammaproteobacteria This study		
Edwardsiella piscicida	Lab isolate		
Salmonella typhimurium	ATCC14028, Lab stock		
Salmonella epidermidis	Lab isolate		
Aeromonas hydrophila	Lab isolate		
Pseudomonas aerugninosa	Lab isolate		
**Genes**			
Toll-like receptor 2 (*tlr2*)	NM_212812.1	tlr2-F	TCTCCGTCTTGGTTTCAC
		tlr2-R	GGTCCCACAGTTGAGTATG
Toll-like receptor4 (*tlr4*)	AY388400.1	tlr4-F	GGAATAATGGGCAGCCGTAAG
		tlr4-R	AGCGACACCAGGAACTATCAATG
Toll-like receptor5a (*tlr5a*)	AY389449.1	tlr5a-F	ACTCCGCTGTTGCTTTGA
		tlr5a-R	GTTTAGACACCACGCAAATGG
Toll-like receptor5b (*tlr5b*)	BC163185.1	tlr5b-F	GAAACATTCACCCTGGCACA
		tlr5b-R	CTACAACCAGCACCACCAGAATG
Myeloid differentiation primary response factor 88 (*myd88*)	DQ100359.1	myd88-F	AACAACTTCGCTGGATAA
		myd88-R	GTTACTGGAATCGCCTCA
Interleukin-1 β (*il1* β)	AY340959.1	il1 β-F	TCAAACCCCAATCCACAGAG
		il1 β-R	TCACTTCACGCTCTTGGATG
ZF NF-kappaB inhibitor alpha-like protein B (*nfkbia*)	AY163841.1	nfkbia-F	GGGTTGGATTCGTTAAAAG
		nfkbia-R	CGTGGATAATGGCGAGATGTAG
Tumer necrotsis factor-α(*tnfα*)	AY427649	tnfα-F	AGAAGGAGAGTTGCCTTTACCGCT
		tnfα-R	AACACCCTCCATACACCCGACTTT
Interleukin-6 (*il6*)	JN698962.1	il6-F	TCAACTTCTCCAGCGTGATG
		Il6-R	TCTTTCCCTCTTTTCCTCCTG
Interleukin-10 (*il10*)	AY887900.1	il10-F	CCCTATGGATGTCACGTCATG
		il10-R	CATATCCCGCTTGAGTTCCTG
Transforming growth factor β (*tgfβ*)	XM_687246	tgfβ-F	CCCAAGGAACCAGAAGTAGAAG
		tgfβ-R	GGATCTTCTATGGTGTGCTGAA
Mucin 2.1 (*muc2.1*)	NC_007136.7	muc 2.1-F	AATATGCCTTGCGGAACAAC
		muc 2.1-R	GTGCTGAGGTTGCAGAATGA
Mucin 5.1 (*muc5.1*)	XM_009297795.1	muc 5.1-F	TGGCAACTTGGCTGATGATA
		muc 5.1-R	TCGTCACACGGACCAGTAGA
Mucin 5.2 (*muc5.2*)	XM_009297793.1	muc 5.2-F	GGTGTCTGTTCCGATCAATC
		muc 5.2-R	TCATCCTTGTCGCCATTGTA
Mucin 5.3 (*muc5.3*)	ENSDARG00000089847	muc 5.3-F	GGGGAAAACTACACCAGCAA
		muc 5.3-R	TGTGAATTCTGTGCCAGAGC
Intestinal alkaline phosphatase1 (*alp1*)	JX847416.1	alp1-F	GTTCCCTTAGAATCCGAGACAC
		alp1-R	GGCCACATAGTTCTGCTCTT
Intestinal alkaline phosphatase 2 (*alpi.2*)	JX847417.1	alpi.2-F	CAGCAGGATTCGATGGATGT
		alpi.2-R	ATTGAGAGCGATGCGTGTAG
Intestinal alkaline phosphatase (*alpi3*)	JX847418.1	alp3-F	ATCTCTTCCAGGGCGTTTATG
		alp3-R	CAGTGGATGTGTCTGGGTTT
Beta defensin β1 (*def β1)*	NM_001081553.1	defb1-F	CTGCTTGTCCTTGTCGTACT

		defb1-R	GCAAACACACTCCTTGTCTG
Beta defensin β2 (*def β2)*	NM_001081554.1	defb2-F	CTTCATTACATTGCCTGCACTT
		defb2-R	GTACCCACACGTCCAATTCT
Beta defensin β3 (*def β3)*	NM_001081555.1	defb3-F	CCAATGATACAGACGTGCAGAG
		defb3-R	GCAGCCACGATAACCAATCA
Heat shock protein (*hsp70*)	NM_002154.4	hsp70-F	GACCTGCCAATCGAGAATCA
		hsp70-R	CCGCTCCTTCTCCAGTTTATC
Heat shock protein (*hsp90a.a*)	NM_131328.1	hsp90a.a-F	CATCGCTAAATCTGGCACAAAG
		hsp90a.a-R	GCCACCAGATACGCAGAATAA
Heat shock protein (*hsp90ab1*)	NM_131310.3	hsp90ab1- F	GAAGAGGAGAAGGCAGAGAAAG
		hsp90ab1-R	CGAGCCGACATCTTCAATCT
CDGSH iron-sulfur domain (*cisd*)	NM_131710.2	cisd-F	GAACCCTCAGACGAACACTAAG
		cisd-R	GGGAAGCCCAGGTTGTATTT
Catalase	NM_001752.4	catalase-F	AGGGTGGTGCTCCAAATTAC
		catalase-R	TGTTGAATCTCCGCACTTCTC
Superoxide dismutase (*sod1*)	NM_011434.2	sod1-F	GGTTCCACGTCCATCAGTATG
		sod1-R	GTCTCCAACATGCCTCTCTTC
β- actin	AF025305	β actin- F	AATCTTGCGGTATCCACGAGACCA
		β actin- R	TCTCCTTCTGCATCCTGTCAGCAA

Table S2. Analytical Profile Index (API) 20E and 20NE biochemical test results of *P. xiamenensis*

***P. xiamenensis*: API 20E**

Test	Results
β -galactosidase	-
Arginine dihydrolase	-
Lysine decarboxylase	-
Ornithine decarboxylase	-
Citrate utilization	-
H_2S production	-
Urease production	-
Tryptophan deaminase	-
Indole production	-
Voges-Proskauer reaction	+
Gelatinase production	+
Glucose	-
Mannitol	-
Inositol	-
Sorbitol	-
Rhamnose	-
Saccharose	-
Melibiose	-
Amygdalin	-
Arabinose	-

***P. xiamenensis*: API 20NE**

Test	Results
Potassium nitrate	+
Tryptophan	+
Glucose (fermentation)	-
Arginine	-
Urea	-
Aesculin	+
Gelatine	+
pNPGb	-
Glucose (Utilization)	+
Arabinose	+
Mannose	+
Mannitol	+
N-Acetyl-glucosamine	+
Maltose	+
Gluconate	+
Caprate	-
Adipate	-
Malate	-
Citrate	-
Phenyl-acetate	-
